# Histone demethylase KDM5B catalyzed H3K4me3 demethylation to promote differentiation of bone marrow mesenchymal stem cells into cardiomyocytes

**DOI:** 10.1007/s11033-022-07428-8

**Published:** 2022-07-05

**Authors:** Zhen Wang, Chenlu Zhong, Hongxiao Li

**Affiliations:** 1 Medical College of Yangzhou University, Yangzhou, 225001 Jiangsu China; 2 Friendliness Hospital Yangzhou, Jiangsu, 225009 China; 3 Department of Cardiology, Northern Jiangsu People’s Hospital, Yangzhou, 225001 Jiangsu China

**Keywords:** KDM5B, H3K4me3, Epigenetic modification, Bone marrow mesenchymal stem cells, Myocardial differentiation

## Abstract

**Background:**

Studies have shown that histone H3 methylation is involved in regulating the differentiation of Bone Marrow Mesenchymal Stem Cells (BMSCs). KDM5B can specifically reduce the level of histone 3 lysine 4 trimethylation (H3K4me3), thereby activating the expression of related genes and participating in biological processes such as cell differentiation, embryonic development and tumor formation. Whether KDM5B is involved in the regulation of BMSCs differentiation into cardiomyocytes through the above manner has not been reported.

**Objective:**

To investigate the effect of KDM5B on the induction and differentiation of swine BMSCs into myocardial cells in vitro.

**Methods:**

Swine bone marrow BMSCs were isolated and cultured, and the overexpression, interference expression and blank vector of KMD5B were constructed and transfected by lentivirus. BMSCs was induced to differentiate into cardiomyocytes by 5-azacytidine (5-AZA) in vitro, and the differentiation efficiency was compared by immunofluorescence, RT-PCR, Western Blot and whole-cell patch clamp detection.

**Result:**

Compared with the control group, the expression levels of histone H3K4me3 and pluripotency gene Nanog in KDM5B overexpression group were significantly decreased, while the expression level of key myocardial gene HCN4 and myocardial marker gene α-Actin and cTNT were significantly increased, and the Na^+^ current density on the surface of differentiated myocardial cell membrane was significantly increased. Meanwhile, the corresponding results of the KDM5B silent expression group were just opposite.

**Conclusions:**

It indicated that enhanced KDM5B expression could promote the differentiation of BMSCs into cardiomyocytes and improve the differentiation efficiency by controlling H3K4 methylation levels.

## Background

Along with the change of environment and the aggravation of population aging, cardiomyocyte damage diseases caused by various pathophysiological factors, including but not limited to heart failure and myocardial infarction, have become an important global health problem. Despite the continuous improvement of clinical diagnosis and treatment measures, the increasing incidence and mortality of cardiovascular diseases still make them one of the most important diseases affecting people's quality of life [1]. Due to the irreversibility of myocardial injury, the clinical efficacy of current therapy for heart failure caused by myocardial injury is very limited [2]. Therefore, it is particularly important to find new way to improve the cardiac function after myocardial injury and reduce the incidence of heart failure.

Stem cell replacement therapy is a promising method for repairing damaged myocardium. A number of studies have confirmed that embryonic stem cells, induced pluripotent stem cells, mesenchymal stem cells (MSC) and other types of stem cells have the ability to differentiate into cardiomyocytes after specific induction [3, 4]. In recent years, several studies have focused on the establishment of induction system for differentiation of stem cells into cardiomyocytes, including 5-azacytidine (5-AZA) induction, co-culture of cardiomyocytes, etc. [5, 6], to explore its regulatory mechanism and clinical application prospects. However, currently commonly used induction methods have defect of low differentiation efficiency and poor stability, which is also the main bottleneck limiting their clinical application [7]. The main reason is that the mechanism of inducing differentiation of stem cells into cardiomyocytes has not been completely clarified. Therefore, to further explore the regulatory mechanism of stem cell induced differentiation into cardiomyocytes through basic research is the theoretical basis and inevitable demand for improving the efficiency of stem cell differentiation, stabilizing the performance after differentiation and promoting clinical application.

The differentiation process of stem cells into cardiomyoid cells is similar to the process of heart development in embryo, which is a complex process involving multi-gene participation and multi-stage accumulation. The sequential activation and expression of related genes at specific time points is the biological basis of embryo development [8]. The sequential activation of the gene, in addition to the gene sequence, is largely determined by the epigenetic modifications. Without affecting the gene sequence, epigenetic modifications affect the binding of transcription factors to the DNA sequence by altering the base or chromatin structure. Thus, the cell development and differentiation can be carried out [9].

Previous studies have found that epigenetic mechanisms involved in the regulation of stem cell differentiation include histone methylation, DNA methylation and histone acetylation [10]. Histone methylation is occurred in the histone N-terminal lysine (K) or at the end of the arginine (R) residues on the methyl group, is mainly regulated by histone methyltransferase (HMTs) and histonelysine (K) demethyltransferases (KDMs). KDM5B can specifically reduce the methylation level of histone 3 lysine 4 (H3K4) without causing changes of other sites, and participate in a variety of biological processes such as embryo development, cell differentiation and tumor formation [11]. At present, studies on KDM5B are mainly focused on embryonic development and tumor formation, but few focus on the regulation of adult stem cells. In this study, swine bone marrow BMSCs were transfected with lentivirus vector to change the expression level of KDM5B. Then 5-AZA was used to induce BMSCs differentiate into myocardium to analyze the role of KDM5B in this process.

## Materials and methods

Experimental animals: 5 Bama miniature swine, weighing 1200–1500 g, 3–5 days old, male or female, provided by Wujiang Tianyu Biotechnology company (License Number SCXK 2016–0006).

Experimental reagents and instruments: lentivirus vectors (up-regulated expression of KDM5B, silenced expression of KDM5B and negative control) with EGFP fluorescent markers were purchased from Nanjing Shengji Biotechnology company (Nanjing, China); GAPDH, KDM5B, H3K4me3, α-actin and cTnT antibodies (Abcam, USA); HCN4 antibody (Nanjing Baode Biotechnology, China); Fetal bovine serum (Gibco, USA); DMEM/F12 medium (Gibco, USA); Polybrene (Sigma, USA); RT-PCR kit (Dalian Bao Bioengineering, China); Fluorescence quantification Kit (QIAGEN, Germany); Western Bolt kit (Wuhan Boshide, China); Patch-clamp amplifier (AXon-700B, USA); Signal acquisition application digital-to-analog Converter (Digidata 1440A, USA); Processing software (PCLAMp 10.4, USA); Microelectrode drawing instrument (P-83, Japan).

### Experimental methods

1. *BMSCs isolation, culture and lentivirus transfection* Small swines were killed by intravenous injection of pentobarbital (85 mg/kg). The bone marrow cavity of bilateral femurs and tibia was rinsed with complete medium (DMEM/F12 medium containing 10% fetal bovine serum). Then cells in the rinse solution were inoculated in 25 cm^2^ flask at a density of 2 × 10^6^/cm^2^. All cells were cultured in DMEM/F12 complete medium containing 10% fetal bovine serum, 100 kU/L penicillin and 100 mg/L streptomycin at 37 °C with 5% CO_2_. The cells were divided into four groups: The up-regulated expression group (KDM5B-ov), silenced expression group (KDM5B-si), negative control group (KDM5B-nc, lentivirus without vector) and Blank control group (no transfection). BMSCs were transfected with the corresponding virus vector in the presence of 6 μg/mL Polybrene for 12–14 h. Following treatment, the cells were observed daily through the use of a fluorescent microscope. The culture medium was replaced 12–14 h after transfection, and cells were collected 72 h later. RT-qPCR demonstrated the strongest interference activity with respect to KDM5B-si. Similarly, KDM5B-ov was screened for the highest expression intensity. Thus, transfected cells were selected for further study.

2. *Immunofluorescence detection of virus transfection efficiency* the third generation BMSCs cultured 72 h after transfection were digested by pancreatin and inoculated in a confocal small dish incubator with a cell concentration of 5 × 10^6^/L. After 24 h, the expression intensity of EGFP was observed by confocal microscope to calculate the transfection efficiency.

3. *BMSCs induced differentiated into myocardium* BMSCs in good condition were inoculated into 12-well plates. When the cell proliferation reached 80% of the area, induction solution (10umol/L 5-AZA + 0.1ug /L CT-L serum-free medium) was added for 24 h. After that, DMEM/F12 medium including 10% fetal bovine serum was used for culture at 37 °C and 5% CO_2_, and the cell morphology was observed every another day.

4. *Immunofluorescence Staining* The isolated BMSCs also known as treated cells were rinsed 3 times with PBS for 10 min each. 4% paraformaldehyde was then added to the cells for a 30-min fixation before paraformaldehyde was absorbed and discarded and rinsed 3 times with PBS. 0.2% TritonX-100 was added to the membrane for permeabilization for 20 min. This was then followed by rinsing 3 times with PBS and treatment with 10% FBS for blocking for 2 h. Blocking solution was extracted and discarded before the addition of primary antibodies α-actin and cTnT proteins in different wells, respectively, overnight. Subsequently, the corresponding secondary antibody was added and incubated for 2 h in the dark, rinsed in PBS for 3 times, and stained with DAPI for 2 min.

5. *RT-PCR evaluation of gene expression levels* At 7, 14 and 21 d of induced differentiation, logarithmic growth cells were taken for trypsin digestion. Cells were collected after 1200 r/min centrifugation for 5 min. Total RNA was extracted by Trizol method and cDNA was synthesized by reverse transcription for RT-PCR. Amplification conditions: 50 °C for 2 min, 95 °C for 10 min, 95 °C for 30 s, 60 °C for 30 s, 40 cycles. Finally, the expression of KDM5B, Nanog and HCN4 in each group were calculated. The experiment was repeated three times. Primer sequences of RT-PCR were shown in Table 1.

**Table 1:** Primer sequences used for RT-PCR

	Primer sequence
GAPDH-F	5′-GGA GTC AAC GGA TTT GGT-3′
GAPDH-R	5′-GTG ATG GGA TTT CCA TTG AT-3′
KDM5B-F	5′-GAA TTC GGG AAT CTT AAA TTT G-3′
KDM5B-R	5′-TAT CTC GAG TTC CTG TTC GGA ATA GG-3′
Nanog-F	5′-GGT TGA AGA CTA GCA ATG GTC TGA-3′
Nanog-R	5′-TGC AAT GGA TGC TGG GAT ACT C-3′
HCN4-F	5′-GTA CTC CTA CGC GCT CTT CA-3′
HCN4-R	5′-GCT CTC CTC GTC GAA CAT CT-3′

6. *Western blot evaluation of protein expression levels* 21 days after induced differentiation, cells in each group were collected, RIPA cell lysate was added, supernatant was centrifuged, concentration was determined by BCA protein concentration assay box and quantitative analysis was performed. Sample loading was 5 μg for each sample. After separation by 10% SDS-PAGE electrophoresis, the protein bands were transferred to PVDF membrane and sealed with 5% skim milk powder for 2 h. The primary antibody (1:500) was incubated at 4 °C overnight, and the secondary antibody (1:2000) was incubated at room temperature for 2 h. After cleaning, the protein bands were colored.

7. *Flow cytometry* Subsequent to treatment with 5‑AZA, the expression of α-actin and cTnT was detected in three groups on different days. Resuspension was done with sterile PBS to adjust the concentration to 2 × 10^8^/L. Preparation was then done by adding 1 × 10^6^ cells into individual tubes incubated overnight at 4 °C with anti‑swine α-actin and cTnT. Then, swine anti‑swine fluorescein isothiocyanate (PE)‑labeled mouse anti‑swine IgG was used as the secondary antibody and incubated with the cells for 1 h at room temperature. The percentage of fluorescent protein‑positive cells was detected by flow cytometry using a BD FACSCanto™ II flow cytometer (BD Biosciences). The results were analyzed and processed by FlowJo version 10.0 (FlowJo LLC).

8. *Tyrode's solution* was composed of NaCl 135 mmol/L, KCl 5.4 mmol/L, NaH_2_PO_4_ 0.33 mmol/L, MgCl_2_ 1.0 mmol/L and HEPES 5.0 mmol/L, CaCl_2_ 1.8 mmol/L, Glucose 10.0 mmol/L, and NaOH were used to adjust pH to 7.3. Calcium-free Tyrode solution and 0. 20 mmol /L Ca^2+^ Tyrode solution were Tyrode solution without CaCl_2_ and 0. 20 mmol/LCaCl_2_, respectively.

The electrode fluid was CsCl 133.0 mmol/L, NaCl 5.0 mmol/L, TEACl 20.0 mmol/L, EGTA 10.0 mmol/L, HEPES 10.0 mmol/L, MgATP 5.0 mmol/L, CsOH was used to adjust pH to 7.25–7.30;

The extracellular fluid was NaCl 135.0 mmol/L, CsCl 5.4 mmol/L, MgCl_2_ 1.0 mmol/L, CaCl_2_ 1.8 mmol/L, HEPES 5.0 mmol/L, Glucose 10.0 mmol/L and CdCl_2_ 0.1 mmol/L, adjust pH to 7.30–7.40 with NaOH.

The KB solution was KOH 110 mmol/L, Taurine 10 mmol/L, Oxalic Acid 10 mmol/L, glutamic acid 70 mmol/L, KCl 25 mmol/L, KH3PO4 10 mmol/L, EGTA 5 mmol/L, HEPEs 5 mmol/L, glucose 10 mmol/L, pH 7.4 adjusted with KOH.

9. *Whole-cell Patch Clamp Detection* Glass microelectrodes with tip diameters of about 1.5–2 μm were prepared by p-83 programmed horizontal drawing instrument. The inlet resistance is maintained at 2–4 Mω, and the series resistance compensation is 30–50%. The cells were placed in extracellular solution and left for 10 min. After adherence, extracellular solution was applied to remove the remaining KB solution. Cells with good condition, smooth edge, integrity, clear horizontal lines, strong three-dimensional sense and no shrinkage were selected for the experiment, and the changes of Na current intensity on cell membrane surface were recorded. In order to minimize the experimental error caused by cell size, current density was used to represent the current value, in pA/pF.

10. Observation indicators (1) Differences in BMSCs lentivirus transfection efficiency and expression levels of KDM5B, H3K4me3, H3K4, Nanog, HCN4, α-actin and cTnT in each group; (2) Differences in cell membrane surface current density of BMSCs in each group.

## Statistical analysis

SPSS 21.0 statistical software was used for statistical analysis. Data were expressed as X ± S. One-way anOVA was used for comparison of cell subsets, one-way ANOVA was used for comparison of mean values between groups 3 or more, and LSD-T test was used for intra-group multiple comparison. All the statistical hypothesis tests were two-sided hypothesis tests, and *p* < 0.05 was considered as significant difference.

## Result

### Morphological changes of BMSCs during induction and differentiation

**Fig. 1:**
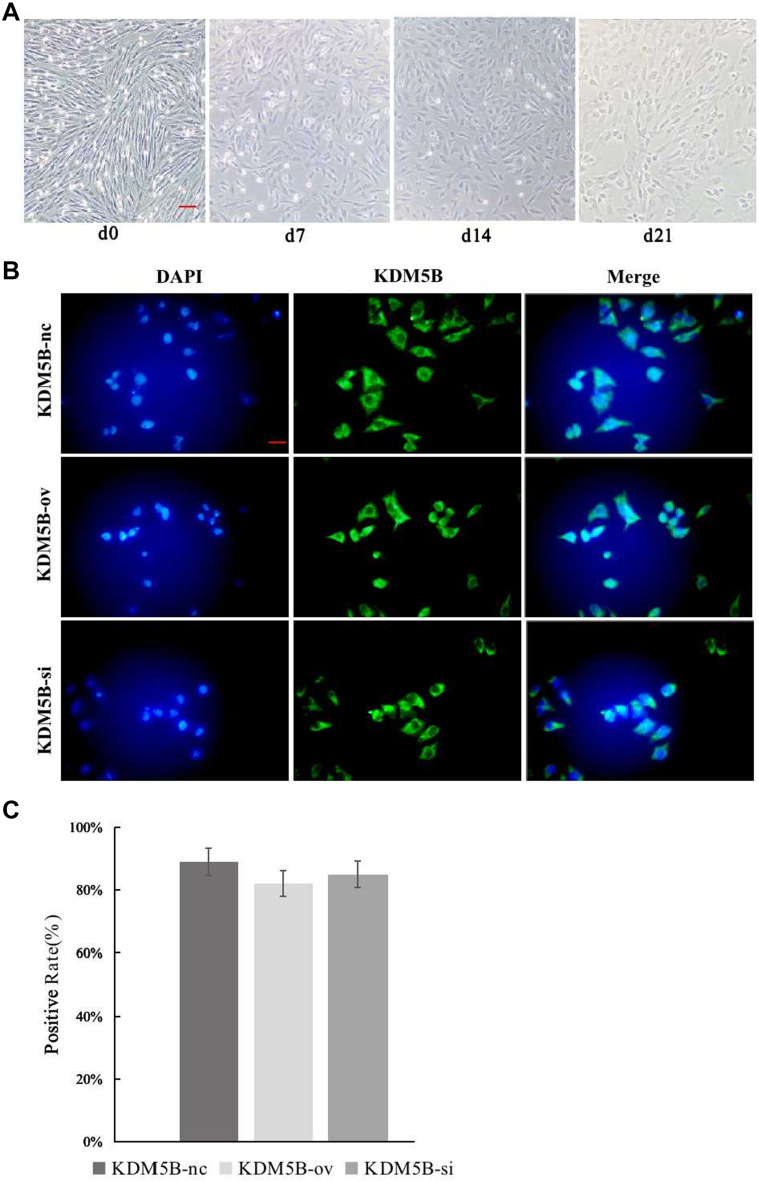
Morphology of BMSCs during differentiation and transfection efficiency of KDM5B. **A**. Morphology of P3 MSCs induced with 5‑AZA for 21 days (× 100, bar = 10 um). **B**. Immunofluorescence was used to detect transfection of lentivirus in the cells. Green fluorescence indicates positive transfection. (× 200, bar = 20 um); **C**. Transfection efficiency calculated according to DAPI and EGFP green fluorescence. There was no significant difference among three groups

Under the microscope, BMSCs with good growth condition grew intensively and appeared as fusiform. 7 days after induction, the morphology of some cells became wider, and the volume increased gradually. 14 days after induction, cell growth slowed down and retraction became short. Cell appears aggregation cluster with short rod-like structure and nucleus in the center, and the ratio of nucleus to plasma decreased significantly, as shown in Fig. 1.

### KDM5B lentivirus transfection efficiency

72 h after stable transfection of lentivirus, confocal laser was used to observe the transfection efficiency. The transfection rate could be estimated according to DAPI blue fluorescence and EGFP green fluorescence under the microscope. There was no significant difference in the transfection efficiency of BMSCs in each group, as shown in Fig. 1.

### Cardiomyocytes differentiation of the MSCs with 5‑AZA induction

**Fig. 2:**
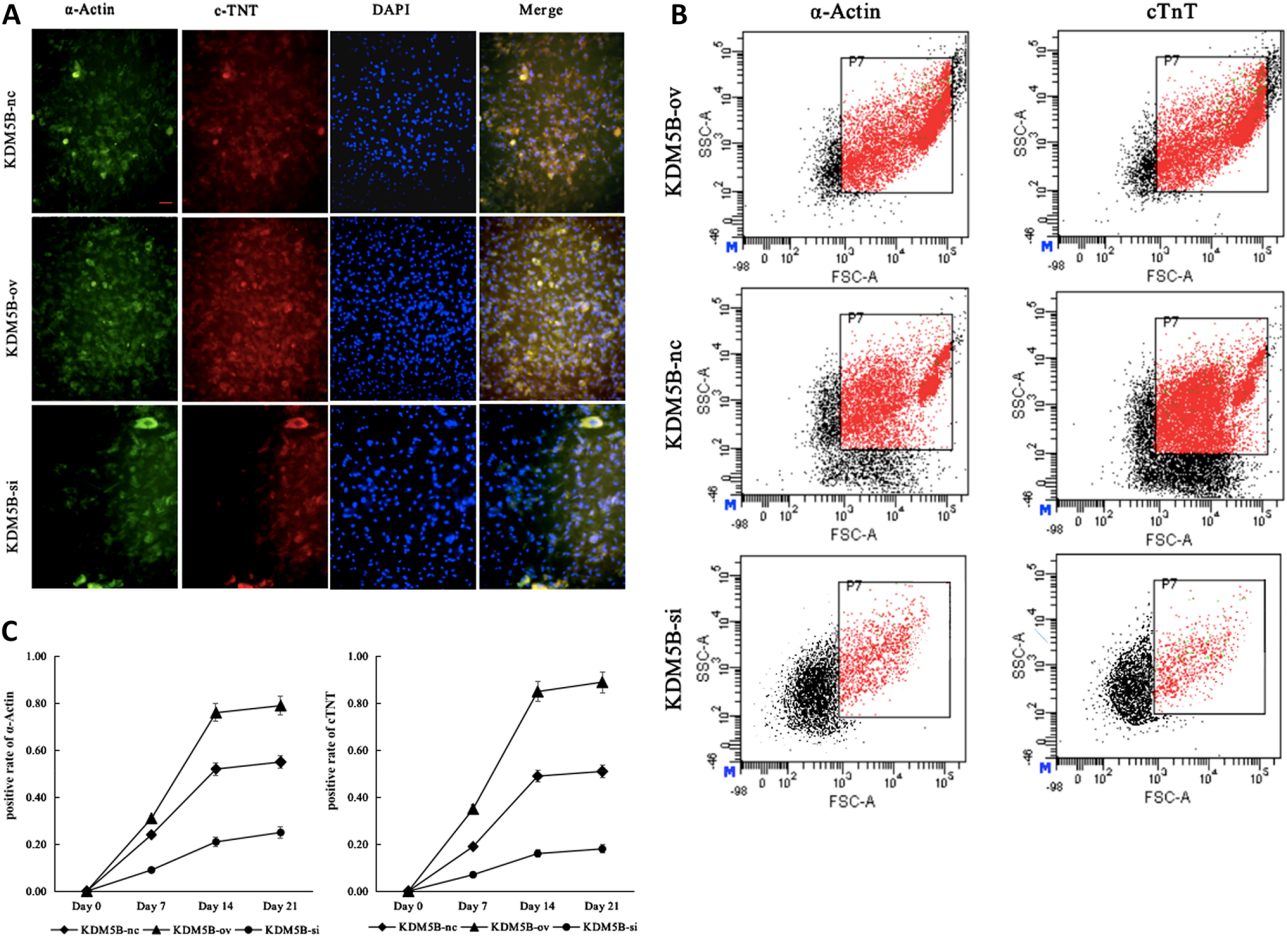
Induction of myocardial differentiation into BMSCs in vitro. **A**. Immunofluorescence staining was used to detect cardiomyocyte marker proteins α-Actin and cTnT expression in cells; green fluorescence indicated the positive expression of α-actin while red fluorescence indicated the positive expression of cTnT (× 100, bar = 10 um). **B**. Fluorescence intensity was detected by means of flow cytometry on day 21, which demonstrated that the proportion of α-Actin- and cTnT‑positive cells rate was ~ 80% in KDM5B-ov group, ~ 50% in the KDM5B-nc group and ~ 20% in the KDM5B-si group. **C**. Flow cytometric analysis demonstrated the percentages of α-actin- and cTnT-positive cells on different days, which demonstrated that α-Actin- and cTnT‑positive cell rate in the KDM5B-ov group was the highest and the KDM5B-si group was the lowest. The mean ± standard deviation is shown; *n* = 3 independent experiments

3. To evaluate the differentiation of BMSCs into cardiomyocytes, the expression of specific proteins known to be important for cardiomyocyte formation and function was investigated. Immunofluorescence staining indicated that BMSCs were strongly positive for cardiomyocyte markers, including α-actin and cTnT. Furthermore, the flow cytometric analysis revealed that the number of α-actin- and cTnT-positive cells began to rise at day 7 and peaked at day14 post treatment, showing a significant difference among three groups. BMSCs α-actin- and cTnT-positive expression rates reached 78.41 ± 3.52% vs 52.37 ± 2.67% vs 19.43 ± 0.87% and 65.38 ± 3.52% vs 45.37 ± 2.47% vs 17.53 ± 0.57% at day 14, respectively. However, there was no significant difference between day 14 and 21 within each group (*p* > 0.05), as shown in Fig. 2. These changes suggest that after upregulation of KDM5B, the differentiation efficiency of BMSCs was raised by approximately 50%. The opposite result was found when KDM5B was downregulated.

### mRNA expression levels of KDM5B, Nanog and HCN4

**Fig. 3:**
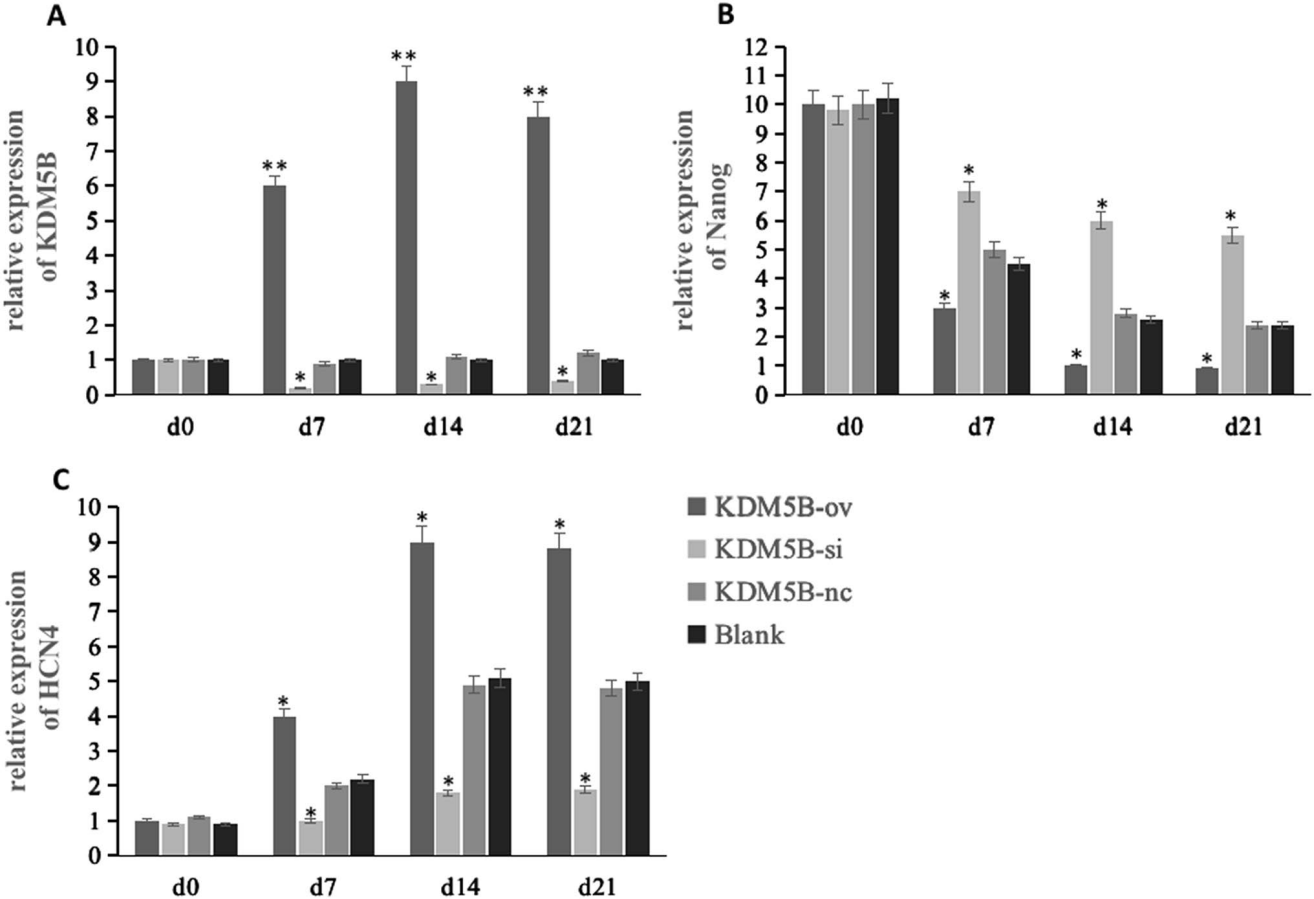
Gene expression levels in BMSCs differentiated into cardiomyocytes. Expression of **A** KDM5B, **B** Nanog and **C** HCN4 was detected via qRT-PCR. The gene expression levels of each group (KDM5B-ov, KDM5B-si and KDM5B-nc) were compared to the blank group (level of significance *p < 0:05; **p < 0:01)

To evaluate changes in gene expression in BMSCs differentiated into cardiomyocytes, we assessed expression of stem cell-specific and cardiomyocyte-specific genes by qPCR. After treatment with 5-AZA, BMSCs continued to proliferate and differentiate. Compared with Blank group, in KDM5B-ov group, the expression of Nanog was significantly decreased (*p* < 0.05) while that of HCN4 was increased (*p* < 0.01). The KDM5B-si group showed the opposite result (*p* < 0.05), while there was no significant difference between KDM5B-nc and Blank group, as shown in Fig. 3. These results indicated that BMSCs differentiation was significantly enhanced after KDM5B overexpression, and BMSCs differentiation was inhibited by KDM5B silencing, but BMSCs differentiation was not significantly affected after lentivirus transfection.

### Changes in protein expression levels of KDM5B, H3K4me3, H3K4 and HCN4 during differentiation

**Fig. 4:**
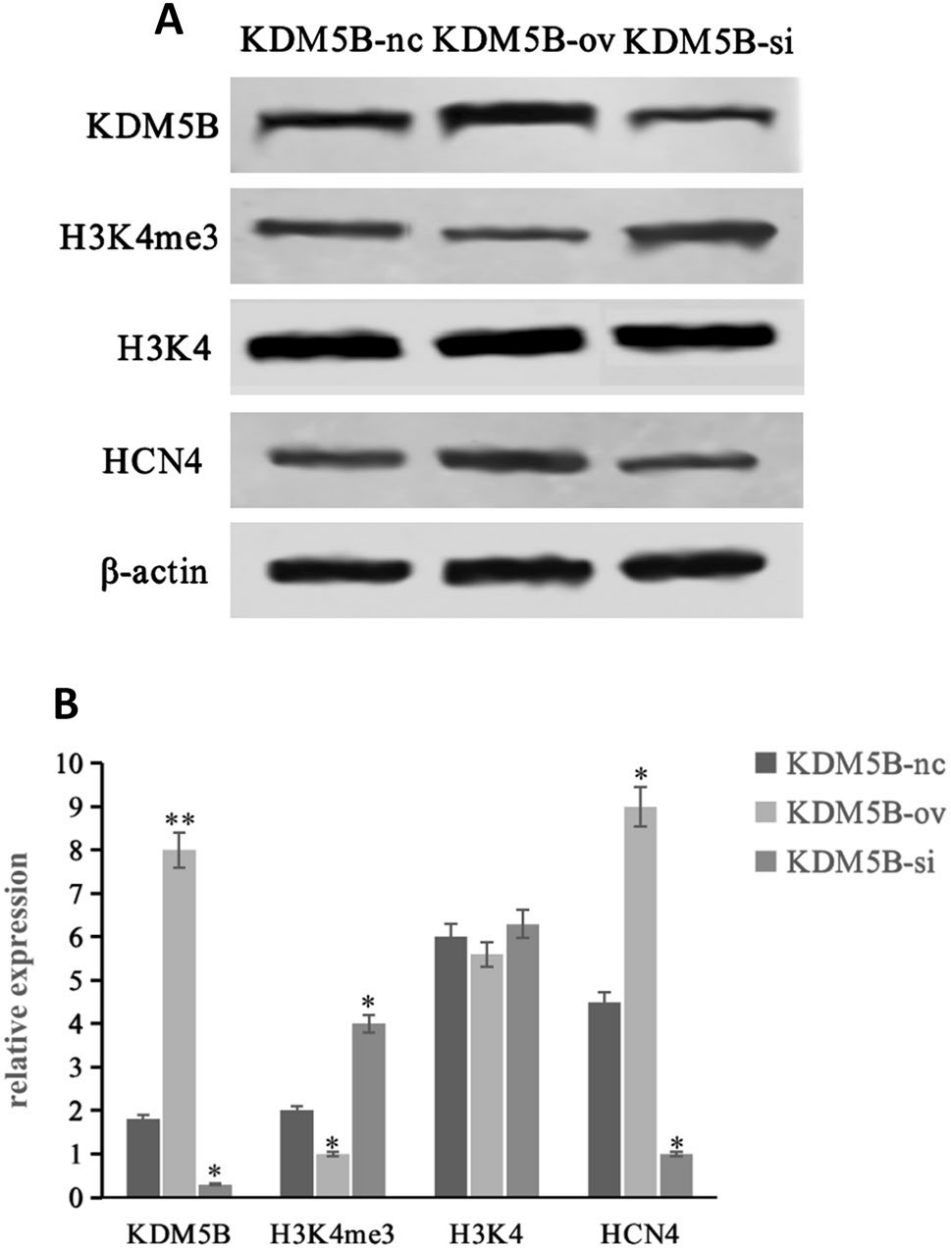
Relative expression levels of protein in BMSCs differentiated into cardiomyocytes. **A**. Following the differentiation of BMSCs, western blotting demonstrated the expression of KDM5B, H3K4me3, H3K4 and HCN4 protein expression, which demonstrated H3K4me3 was downregulated while HCN4 was upregulated following KDM5B upregulation. H3K4 and β-actin was used as an internal control. **B**. Cardiomyocyte differentiation of the BMSCs was detected by RT-qPCR, which demonstrated that KDM5B upregulation increased the expression of HCN4 by 2 times compared with the control group. The opposite result was found when KDM5B was downregulated. *p < 0.05 and ** *p* < 0.01 vs. the KDM5B-nc group

In order to compare the effect of KDM5B on the final induction efficiency, the expression of protein H3K4me3, H3K4 and HCN4 was detected by means of western blotting and RT‑qPCR. Western blot showed that, compared with KDM5B-nc group, in KDM5B-ov group, the relative expression level of H3K4me3 protein was significantly decreased (*p* < 0.05), and that of HCN4 was significantly increased (*p* < 0.01), while the KDM5B-si group was opposite (*p* < 0.05), as shown in Fig. 4. The results demonstrated that the reduction of H3K4me3 by interfering with KDM5B could increase the efficiency of the BMSCs induced differentiation into cardiomyocytes. Further more, these results also suggested that regulation of HCN4 expression way one of the pathways through which KDM5B played its role.

### Comparison of sodium current density on cell membrane surface

**Fig. 5:**
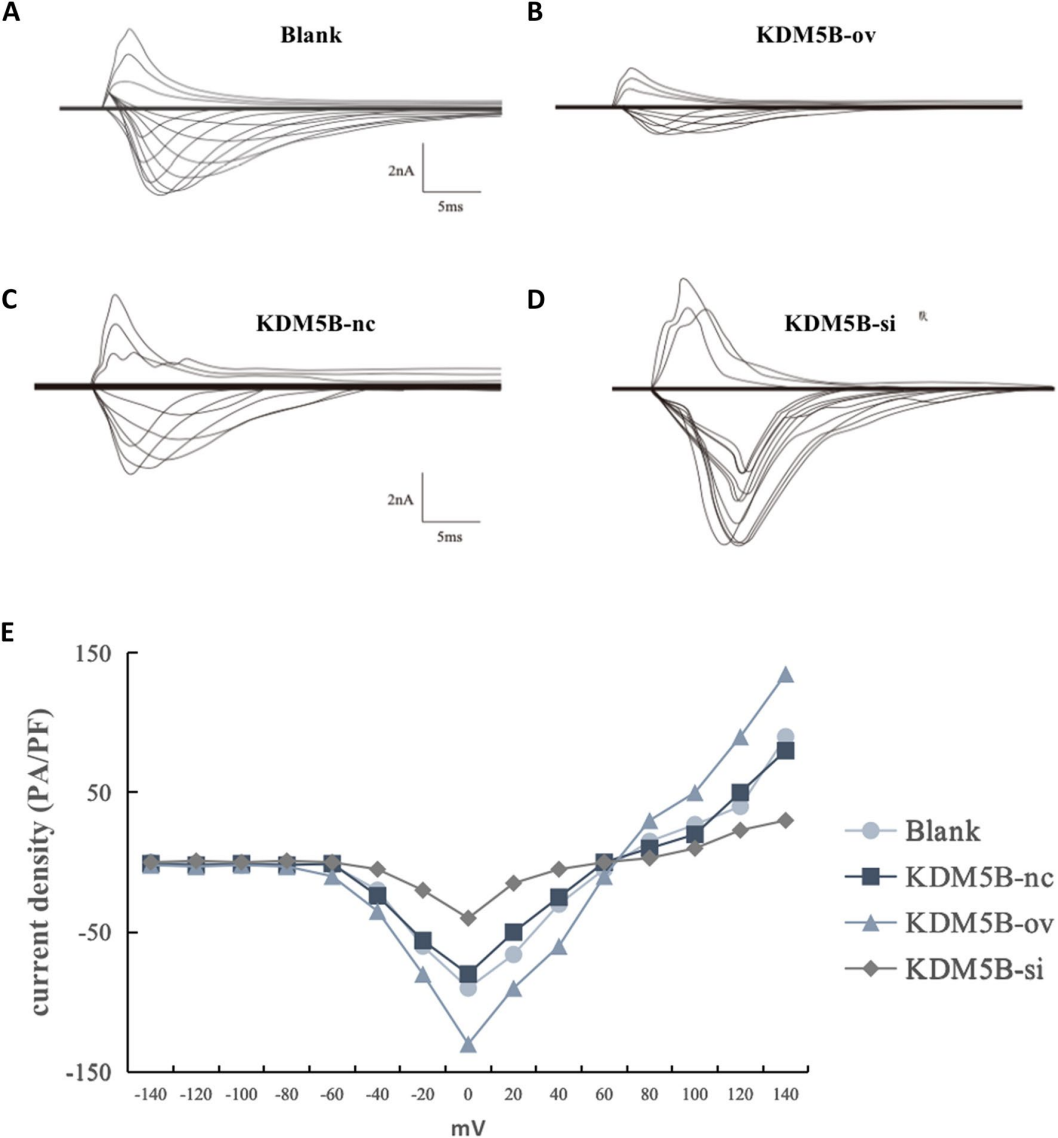
Density of INa current in each group of cells. Whole-cell patch clamp was used to detect Na current density on cell membrane surface and the results showed that KDM5B overexpression (**B**, **E**) increased the sodium current density compared with the control (**A**, **C**, **E**). The opposite result was found when KDM5B was downregulated (**D**, **E**)

Whole-cell patch clamp test results showed that compared with the KDM5B-nc group, the sodium current density of the KDM5B-ov group was significantly increased, while that of the KDM5B-si group was significantly decreased, as shown in Fig. 5, which proved that overexpression of KDM5B could improve the physiological function of cardiomyocytes differentiated from BMSCs.

## Discussion

One of the major interests about stem cells is their potential use for therapeutic applications [12]. So far, several kinds of stem cells, including embryonic stem cells (ESCs), induced pluripotent stem cells (iPSCs) and mesenchymal stem cells (MSCs) are proven to be pluripotent, giving rise to all the cells from the three embryonic germ layers [13, 14]. Evidence has accumulated that both murine and human adult tissues contain early-development stem cells with a broader differentiation potential than other adult monopotent stem cells [15, 16]. The nonhematopoietic stem cells appear to be heterogeneous and contain cells committed to mesenchymal and endothelial lineages, as well as more primitive multipotential cells resembling progenitors of germ cells [17]. Although tremendous progress has been reached in mouse, the physiological traits of rodents are far apart from human. Swine and human are highly homologous, which are treated as the optimal organ donor. The research of MSCs residing in swine bone marrow may expedite possible applications of this intriguing cell in regenerative and precision medicine.

Although there are still considerable dispute in the clinical effect based on stem cells therapy, the positive results obtained in the repair of damaged myocardium indicated it has become a promising candiadate [18]. In fact, diverse stem cells have been identified and applied to the regeneration medicine including bone marrow-derived mononuclear cell (BM-MNCs) [19] and umbilical cord blood-derived stem cells (UCB-SCs) [20]. Studies have shown pluripotent stem cells (PSCs) are precursors of monopotent stem cells during organ/tissue rejuvenation and a source of these cells in emergency situations when organs are damaged (e.g., myocardial infarction or stroke) [21]. The application of PSCs has shown very encouraging results, including iPSCs and epiblast-like stem cells (ESCs) isolated from UCB.

A rare Sca1 + Lin − CD45 − SCs population were initially identified, isolated from adult mice via fluorescence activated cell sorting and named as very small embryonic-like stem cells (VSELs) [22]. VESLs were furtherly isolated from human cord blood [23], several adult murine tissues and organs [24]. Recent reports proposed that VSELs deposited in adults tissue share several markers with epiblast/germ line cells and are responsible for tissue regeneration after organ injuries in rejuvenation of the TCSCs [25]. Besides, evidences demonstrated that that VSELs coming from primordial germ cells, give rise to hematopoietic stem cells (HSCs), and endothelial progenitor cells (EPCs) and are a source of mesenchymal stem cells (MSCs) and TCSCs [26]. As a promising candidate, their unique characteristics and potentiality may contribute to myocardial and endothelial regeneration. Our team has also carried out relevant research on VSELs. Based on our current research results, we believe that there are both VSELs and MSCs in the bone marrow, and VSELs are relatively more potent, showing more primitive stem cell characteristics. There are some differences between these two kinds of cells in terms of extraction methods, culture conditions and morphological characteristics. The morphological changes during induction differentiation were not identical [27, 28].

5-AZA, widely used in the cardiac differentiation of stem cells, can regulate the histone demethylation and DNA Methylation [29]. A number of stem cells have been used to differentiate into cardiomyocytes by 5-AZA [30, 31]. The mechanism of 5-AZA may be related to CpG base-pair demethylation and regulation of early myocardial transcription factors [32, 33]. Currently, the efficiency of stem cell induction into cardiomyocytes is low, which limits its clinical application [34]. In the present study, epigenetics was used to improve the differentiation efficiency of stem cells.

Epigenetic inheritance refers to the fact that DNA sequence does not change, but gene expression has undergone heritable changes, including histone modification, DNA methylation, X chromosome inactivation, non-coding RNA regulation, etc. [35]. The n-terminus of histones is an unstable subunit with no specific structure that extends beyond the nucleosome and is subject to various chemical modifications, especially the n-terminus residues of histones H3 and H4 and the N-terminus and C-terminus of histones HZA and HZB and H1, which can be modified by methylation, acetylation, phosphorylation and ubiquitination. Covalent modification of histone can change the set of interactions between proteins and DNA, nucleosome structure change, change the histone the ability to combine with other proteins, and plays an important role in regulation of chromatin structure, many involved in chromatin biology process are modulated by histone modification, including replication, repair, transcription, maintaining genomic stability, etc. [36–38]

Previous studies have found that KDM5B is involved in cell cycle, mitosis, proliferation, self-renewal and differentiation of embryonic stem cells by changing H3K4 methylation levels related to target gene promotors [11, 39, 40]. However, these studies mainly focus on embryonic development and tumor, with little involvement in the induction and differentiation regulation of adult stem cells. During the differentiation of stem cells into cardiomyoid cells, histone modification enzymes cluster in the transcription regulatory region of cardiac genes, forming histone modification enzyme-transcription factor complex to regulate gene expression and cell differentiation [41]. In this study, different groups of BMSCs were induced into myocardium, and it was found that the expression of HCN4, a key gene of myocardial differentiation, was increased in the KDM5B overexpression group, while significantly decreased in the KDM5B silencing group, which led to the change of cell membrane surface current density after differentiation, changing the physiological function of the differentiated cells. These results suggest that KDM5B may influence the expression of HCN4 at chromatin structure level by regulating the methylation status of HCN4 histone H3K4, regulating the differentiation of BMSCs.

HCN is a superactivated cyclic nucleotide gated cation channel, including HCN1, HCN2, HCN3 and HCN4 subtypes, among which HCN1, HCN2 and HCN4 subtypes are mainly expressed in the heart. HCN gene is a crucial molecular basis for normal cardiac pacing and is mainly distributed in the conduction system of the heart [42]. HCN4 can encode hyperpolarized activated cationic current (If) channel structural proteins and participate in the spontaneous depolarization process of sinoatrial node cells, which is closely related to the generation of cardiac pacing and rhythm regulation, as well as the sympathetic nerve regulation of heart rate [43].

HCN4 gene plays a major role in the spontaneous electrical activity of sinoatrial node pacemakers, but also participates in fetal heart development and maintenance of sinus rhythm after birth. Animal experiments showed that HCN4 gene knockout mice could be lethal at 9.5–11.5d of gestation age, which is the time of sinoatrial node development. Therefore, it was considered that the lethality of the embryo might be related to the undevelopment of sinoatrial node [44, 45]. However, mice with partial deletion of HCN4 gene had significantly reduced heart rate despite normal heart morphology, suggesting that HCN4 is necessary for heart development and normal conduction [46, 47]. After deletion of HCN4 gene or one of the gene loci in adult mice, reduced amplitude of If current can be detected, and repeated cardiac arrest or bradycardia can be observed in surface electrocardiogram [48]. HCN4 is highly expressed in human embryonic stem cells and early cardiac progenitor cells, and the presence of If current can be detected [49]. Our previous study found that transfection of HCN4 gene can promote the differentiation of BMSCs into cardiomyoid cells [27]. In this study, upregulation of KDM5B expression can improve the expression level of HCN4 gene and the differentiation ability of BMSCs into cardiomyoid cells, suggesting that HCN4 gene may be one of the target genes promoting the role of KDM5B. Unfortunately, the interaction mechanism between KDM5B and HCN4 was not further clarified in this study. Therefore, in the next stage of the study, further research will be conducted on this aspect. Besides, the major purpose of this study was to determine whether KDM5B could affect the differentiation process of MSC. So we explored it at the gene, protein and cellular levels and both enhanced and interfered expression vectors were used. Next, on the basis of the existing research results, we will further explore the specific regulatory mechanism of KDM5B, and try to clarify the signaling pathway that KDM5B participates in. Therefore, in the subsequent experimental design, the control would contain the empty backbone vector carrying lentivirus instead of lentivirus without vector.

## Conclusion

Overexpression of KDM5B can promote the differentiation of BMSCs into cardiomyocytes. One possible mechanism is to activate the expression of related genes by catalyzing the demethylation of H3K4me3. The regulation of BMSCs differentiation by epigenetics may be a new method to modify seed cells in tissue engineering.

## Data Availability

All data generated or analyzed during this study are included in this published article.

## Abbreviations

BMSCs: Bone Marrow Mesenchymal Stem Cells
H3K4me3: Histone 3 lysine 4 trimethylation
